# Clinical evidence for fat-binding–mediated fecal fat excretion by RODL^TM^ (Red Okra–*Diospyros lotus* botanical combination) under a prescribed restricted diet: a randomized, double-blind, placebo-controlled trial

**DOI:** 10.29219/fnr.v70.13900

**Published:** 2026-07-04

**Authors:** Soo-Yeon Park, Jiyea Park, Sunoh Kim

**Affiliations:** 1 Department of Ophthalmology, Otolaryngology & Dermatology, College of Korean Medicine, Dongshin University, Naju-si, Republic of Korea; 2 BigsomeBio, Gyeonggi-do, Republic of Korea; 3 Central R&D Center, B&Tech Co., Ltd., Naju-si, Republic of Korea; 4 Department of Biomedical Laboratory Science, Gwangju Health University, Gwangju, Republic of Korea

**Keywords:** red okra, *Diospyros lotus*, Fecal fat excretion, fat-binding agent, prescribed restricted diet, clinical trial

## Abstract

Preclinical studies have demonstrated that a botanical combination of red okra (*Abelmoschus esculentus*) and *Diospyros lotus* (RODL^TM^) exerts dual anti-obesity actions by both inhibiting intestinal fat absorption and stimulating adipocyte lipid metabolism. However, its clinical efficacy in promoting fecal fat excretion under controlled dietary conditions has not been validated in humans. A randomized, double-blind, placebo-controlled clinical trial was conducted in 70 healthy adults under a prescribed restricted diet containing 130–150 g/day of fat. Participants were randomly assigned to receive either RODL (*N* = 34, 1.5 g/day) or placebo (*N* = 36) for 2 days. Fecal samples were collected at baseline and post-intervention to quantify fecal fat content (mg/g wet and dry weight, and total mass). Blood lipid profiles and free fatty acids were also assessed. Data were analyzed using the full analysis set and per protocol set with appropriate statistical tests. Following 2-day administration, the RODL group showed a significantly greater increase in fecal fat excretion compared to the placebo group, as measured by wet fecal fat (28.65 ± 17.46 mg/g vs. 18.54 ± 11.86 mg/g, *P* = 0.0034), dry fecal fat (58.39 ± 29.95 mg/g vs. 39.54 ± 21.39 mg/g, *P* = 0.0025), and total fecal fat mass (3,238 ± 3,636 mg vs. 1,802 ± 1,713 mg, *P* = 0.0118). The botanical combination of RODL significantly enhances fecal fat excretion under a standardized fat-controlled diet without affecting fecal matrix properties or safety parameters. These findings support its potential and safe fat-sequestering agent for dietary fat management.

## Popular scientific summary

- In a randomized, double-blind, placebo-controlled human trial under a prescribed restricted diet (130–150 g fat/day), RODL (red okra + *Diospyros lotus*) increased fecal fat and triglyceride excretion compared with placebo after 2 days.
- The findings support a physical fat-binding mechanism without altering fecal moisture or fecal weight.
- RODL provides clinical evidence for a dietary approach to modulate intestinal fat handling under controlled intake conditions.

The global prevalence of obesity has risen sharply in recent decades due to increased caloric intake and reduced physical activity, driven by Westernized dietary patterns and sedentary lifestyles. Obesity is a multifactorial metabolic disorder characterized by excessive fat accumulation, which elevates the risk of chronic diseases such as type 2 diabetes, dyslipidemia, hypertension, and non-alcoholic fatty liver disease (1–3). Consequently, obesity imposes serious threats to both personal health and societal productivity, primarily due to increased medical demands and reduced workforce efficiency (4). Given the limitations and adverse effects associated with pharmacological interventions, there is increasing interest in safe, food-derived agents that can aid in the regulation of lipid metabolism and energy balance (5).

Among the dietary components, excessive dietary fat intake plays a pivotal role in the development and progression of obesity and related metabolic syndromes. Consequently, strategies aimed at reducing intestinal fat absorption or enhancing fecal fat excretion have garnered increasing interest as viable interventions for obesity management (5). Several natural fibers and phytochemicals have been shown to possess fat-binding properties, thereby limiting intestinal lipid absorption and enhancing fecal fat excretion (6). In particular, soluble dietary fibers are known to form viscous gels in the gastrointestinal tract, which can trap dietary lipids, interfere with micelle formation, or modulate bile acid metabolism, ultimately resulting in decreased fat absorption (7). Moreover, they are fermented by gut microbiota into short-chain fatty acids (SCFAs), including acetate, propionate, and butyrate, which enhance gut barrier function, modulate immune responses, and regulate lipid and glucose metabolism (8, 9). These SCFAs have been associated with improved intestinal barrier function, reduced systemic inflammation, and altered hepatic lipid metabolism. Notably, certain plant-based polysaccharides have demonstrated the ability to reduce cholesterol biosynthesis and insulin demand through SCFA-mediated mechanisms (10).

The global rise in obesity and metabolic syndrome has highlighted the need for non-pharmacological interventions that target excessive dietary fat intake and storage. In this context, plant-derived agents with fat-binding capacity have gained interest for their ability to reduce intestinal lipid absorption and facilitate fecal excretion, thereby attenuating postprandial lipid accumulation. Among plant-derived interventions, those with gastrointestinal fat-binding properties offer dual benefits: reducing intestinal lipid absorption and facilitating fecal fat excretion, thereby limiting caloric intake without systemic side effects (4, 5). Dietary fibers and botanical extracts with lipophilic binding properties have been investigated for this purpose, but few have demonstrated both mechanistic efficacy and clinical tolerability.

Red okra (*Abelmoschus esculentus*, RO) is rich in viscous mucilage, polyphenols, and dietary fiber that may physically interfere with lipid emulsification and micelle formation, thereby limiting fat absorption in the gastrointestinal tract (11). Similarly, *Diospyros lotus* (DL) leaves are known to contain flavonoids such as myricetin and gallic acid derivatives that have been reported to exhibit lipolytic, anti-adipogenic, and antioxidant effects (12). These components have been previously reported to exhibit anti-obesity activity by modulating lipid metabolism and adipocyte differentiation *in vitro* and *in vivo.* Our preclinical studies conducted using the combined extract of red okra and *D. lotus* (RODL) have demonstrated dual anti-obesity mechanisms: suppression of lipid accumulation in adipocytes and enhanced fecal fat elimination through physical binding of dietary lipids in the gastrointestinal tract (13). *In vitro* studies have demonstrated that a RODL significantly inhibits adipocyte differentiation and intracellular lipid accumulation. Furthermore, animal studies have shown that oral administration of the RODL formulation reduces plasma triglyceride (TG) levels while enhancing fecal lipid excretion, indicating a potential fat-blocking mechanism via intestinal sequestration of dietary lipids. In high-fat diet-fed rodents, co-administration of the RODL extract significantly reduced serum TGs and hepatic lipid accumulation, while increasing fecal TG excretion, suggesting both systemic and luminal effects on lipid metabolism. These findings highlight the potential of the RODL combination as a multi-targeted botanical strategy for obesity prevention and metabolic regulation.

However, despite the mechanistic plausibility and preclinical efficacy of this formulation, its functional impact on lipid excretion has not been validated in human trials under controlled dietary conditions. Moreover, fecal matrix variability and dietary intake are critical confounding factors in fecal fat analysis, which must be carefully managed to ensure valid clinical outcomes (8).

To address this, we conducted a randomized, double-blind, placebo-controlled clinical trial in healthy adults under a prescribed restricted diet to investigate the fat-excreting efficacy and safety of RODL. By standardizing dietary fat intake and fecal collection timing, we aimed to isolate the effects of RODL on fecal fat excretion, while simultaneously monitoring its impact on blood lipid profiles and safety parameters. This clinical trial provides the first evidence that RODL significantly enhances fecal fat excretion in humans when administered under standardized dietary conditions.

## Materials and methods

### Study design

**Fig. 1:**
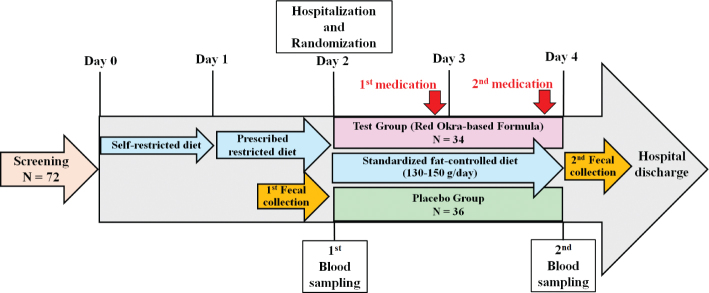
Schematic overview of the clinical study design evaluating the effects of RODL on fecal fat excretion. Participants (*N* = 72) were initially screened, and those eligible were enrolled and randomized into either the test group (RODL, *N* = 34) or placebo group (*N* = 36). To ensure standardized baseline fecal conditions, all participants followed a dual-phase dietary control regimen comprising a 2-day self-restricted diet and a 1-day prescribed restricted diet prior to hospitalization. On Day 1, baseline fecal samples were collected, followed by the initiation of a standardized fat-controlled diet (130–150 g fat/day) throughout a 2-day inpatient period. Participants received the assigned investigational product (RODL or placebo) immediately after dinner on Days 1 and 2. Fecal samples were recollected on Day 3, prior to hospital discharge. This design allowed for controlled assessment of the effects of RODL on fecal fat output under tightly regulated dietary conditions. RODL: red okra (*Abelmoschus esculentus*) and *Diospyros lotus*.

This randomized, double-blind, placebo-controlled, parallel-group clinical trial was conducted to evaluate the efficacy and safety of RODL in enhancing dietary fat excretion via fecal elimination. To minimize inter-individual variability in baseline fecal fat measurements, we established a standardized pre-collection dietary protocol based on findings from a preliminary pilot trial. In this preliminary study (data not shown), we compared participants who had undergone dietary adjustment prior to fecal sample collection with those who had not, and observed substantial within-group variance in fecal fat parameters, likely attributable to uncontrolled dietary fat intake. To address this issue, a dual-phase dietary control was implemented, consisting of a 2-day self-administered restricted diet beginning 2 days before admission, with only the provided restricted meals consumed on the day prior to hospitalization (Fig. 1). This dual-phase dietary control was designed to minimize inter-individual variation by standardizing fat intake prior to fecal collection. The same protocol was applied in the study, ensuring baseline standardization across participants and minimizing between-group differences at Day 2.

### Ethical considerations

The clinical trial was performed at Dongshin University Korean Medicine Hospital in Naju, Republic of Korea, in compliance with the Declaration of Helsinki and the International Council for Harmonisation of Technical Requirements for Pharmaceuticals for Human Use – Good Clinical Practice (ICH-GCP) and approved by the institutional review board (IRB approval number: NJ-IRB-019, protocol number: BAT-BF-BRO1, Protocol Version:2.0, IRB approval date: 29 September 2022). All participants provided written informed consent prior to enrollment.

### Inclusion and exclusion criteria

Participant eligibility criteria for screening and enrollment are presented in Table 1. Briefly, healthy adults aged 19–65 years with a body mass index (BMI) of 20–30 kg/m^2^, regular bowel habits (1–2 times/day), and no history of gastrointestinal disorders were eligible for inclusion. Individuals with significant cardiovascular, hepatic, renal, gastrointestinal, or metabolic conditions; recent use of lipid-lowering agents or dietary supplements affecting fat metabolism, or participation in another clinical trial within 3 months prior to screening were excluded.

**Table 1:** Eligibility criteria for participant enrollment

Category	Description
Inclusion criteria analysis	Healthy adults aged 19–65 years
	BMI of 20–30 kg/m^2^
	Regular bowel habits (1–2 times/day)
	No history of gastrointestinal disorders
	Written informed consent obtained prior to enrollment
Exclusion criteria	Clinically significant cardiovascular, hepatic, renal, gastrointestinal, or metabolic disease
	Recent use of lipid-lowering medications
	Recent use of dietary supplements affecting fat metabolism
	Participation in another clinical trial within 3 months prior to screening

BMI: body mass index.

### Sample size calculation

The primary objective of this study was to evaluate the superiority of the test product (RODL) over placebo in enhancing fecal fat excretion during the intervention period. The statistical hypothesis was defined as follows:

Null hypothesis (H_0_): μ_t_ = μ_⊂_ (no difference in fecal fat excretion between test and control groups)

Alternative hypothesis (H_1_): μ_t_ ≠ μ_⊂_ (fecal fat excretion differs between groups).

This was a two-sided superiority test with the following assumptions:

1. Significance level (α) of 0.05 (two-tailed);
2. Power (1 − β) of 80%, corresponding to a Type II error (β) of 0.20;
3. Equal allocation ratio (1:1) between test and placebo groups;
4. Reference values for mean difference and standard deviation (SD) (Δ = 1.9, σ = 2.4) were derived from a previous clinical study with a similar primary outcome (14);
5. A conservative estimate for the clinically meaningful effect size (Δ) was applied, which is smaller than the reported mean difference of 4.0 g.

Based on the standard sample size formula for comparing two independent means:

*n* = [2 × (Z_a_/2 + Z_β_)^2^ × σ^2^] / Δ^2^

*n* = [2 × (1.96 + 0.84)^2^ × 2.4^2^] / 1.9^2^ ≈ 26 participants per group

Z_a_/2: The critical value or quantile at which the area in the right tail of the standard normal distribution becomes α/2.

Z_β_: The critical value or quantile at which the area in the right tail of the standard normal distribution becomes β.

α: The magnitude of the Type I error.

β: The magnitude of the Type II error.

After accounting for an estimated 25% dropout rate, the final required sample size was determined to be 35 participants per group, totaling 70 participants for the study.

### Participant withdrawal criteria

The completion status of all participants enrolled in the clinical study was documented, and the reasons for any discontinuation of test food intake or study observation were recorded. Participants were withdrawn from the study under the following predefined conditions:

1. If the participant was found to be in violation of the inclusion or exclusion criteria;
2. If a serious adverse event (SAE) or an adverse event (AE) occurred that, in the judgment of the investigator, warranted discontinuation of the study for safety reasons;
3. If a previously undetected systemic disease was identified during the study period;
4. If the participant or legal representative voluntarily withdrew informed consent at any time;
5. If the participant became lost to follow-up and could no longer be contacted;
6. If the participant was unable to consume the test food as required by the study protocol;
7. If the participant self-administered any medications or consumed foods outside the provided restricted diet that could influence study outcomes, without prior approval from the investigator;
8. If the investigator determined that continued participation was not appropriate due to any other clinical or protocol-related reason;
9. If the participant failed to defecate by 2:00 PM on Day 2 or before discharge at 4:00 PM on Day 4;
10. If the participant was confirmed to be pregnant during the study period.

All withdrawals were recorded and included in the Safety Set analysis, and reasons for discontinuation were described in the final dataset and statistical analysis plan.

### Enrollment and randomization

A total of 72 subjects were screened, of whom 70 met all inclusion and exclusion criteria and were randomized to the test group (*n* = 34) or the placebo group (*n* = 36). One participant in the test group was excluded from the full analysis set (FAS) and per protocol set (PPS) sets due to non-compliance with the investigational product intake on Day 2. Accordingly, 69 subjects completed the study and were included in the FA and PP set. Randomization was implemented using a block randomization method with a 1:1 allocation ratio between the intervention group (RODL) and the placebo group. Randomization codes were generated using SAS^®^ Version 9.4. Allocation concealment and blinding were maintained through identical packaging and labeling of investigational products, with no breach of blinding reported throughout the study.

### Preparation of the RODL

The botanical formula used in this study consisted of a mixture of RODL in a 4:1 (w/w) ratio. The formulation ratio and dosing regimen were previously determined through our published preclinical study using cell-based and animal models and were adopted for the present clinical trial. Detailed information on the major active constituents and their quantitative composition has been reported in that earlier publication [13], which is cited here as the primary reference for formulation development and phytochemical characterization. Briefly, authenticated plant materials were subjected to hot-water extraction, followed by filtration, vacuum concentration, and drying to obtain powdered extracts. The resulting extracts were then blended and formulated into the investigational product used in the present clinical trial.

### Dietary instruction

At each study visit, the clinical research coordinator provided standardized dietary guidance to all participants to ensure adherence to the controlled dietary protocol. Participants were instructed to consume only the meals and snacks provided at designated times during the intervention period and to refrain from consuming any additional food or beverages, including alcohol. Furthermore, they were advised to consume the pre-specified meals provided the day prior to hospital admission and to avoid high-fat or greasy foods throughout the study. Compliance with dietary instructions was reinforced verbally at each visit to minimize variability in fat intake. During the hospitalization period, all participants were provided with standardized meals at fixed time windows: breakfast around 9:00 AM, lunch around 12:00 PM, and dinner around 6:00 PM. The test product or placebo was administered within 10 min after the completion of dinner. All meals were identical in composition across participants to ensure consistency in dietary fat intake during the intervention.

### Intervention and dosing

Participants were randomly assigned to receive either the investigational product (RODL) or a matching placebo over a 2-day intervention period during a 2-day inpatient clinical setting. The test product was formulated as a liquid sachet (10 g per dose) containing 1.5 g of a RODL. The remaining 8.5 g consisted of food-grade excipients, including malt extract, citric acid anhydrous, sucralose, apple flavor, and purified water. The placebo matched the test product in appearance, color, viscosity, and taste, but contained no active botanical components. Both products were manufactured under identical conditions and packaged in identical aluminum sachets. The intervention was designed to evaluate the effects of the RODL on fecal fat excretion under controlled dietary conditions. All participants were admitted to the clinical research unit on Day 1 of the intervention period. During the 2-day hospital stay, participants received a controlled fat diet standardized to 130–150 g of fat per day, distributed evenly over three meals per day. To isolate the effect of the intervention on lipid absorption and excretion, no additional foods or beverages were allowed beyond the provided meals and water. Each participant received one dose (10 g sachet) of the assigned investigational product on two consecutive evenings, administered within 10 min after dinner. All administrations were directly supervised by clinical staff to ensure full compliance and to monitor for any immediate AEs. The timing of ingestion relative to meals was controlled to reduce inter-individual variation in gastrointestinal lipid absorption kinetics.

### Physical examination and vital signs

Comprehensive physical examinations were conducted at Days 2 and 3, evaluating the clinical status of participants across multiple organ systems, including the cardiovascular, pulmonary/respiratory, gastrointestinal/hepatobiliary, metabolic/endocrine, renal/urinary, reproductive, musculoskeletal, dermatological/connective tissue, neurological, psychiatric, and other relevant systems. Vital signs, including systolic and diastolic blood pressure (BP) and pulse rate, were measured after participants had rested for at least 10 min in a seated position, and assessments were performed at Days 2, 3, and 4. Anthropometric measurements were also conducted: height and BMI (kg/m^2^) were measured at Day 2, while body weight was recorded at Days 1–3. Height and body weight were measured to the nearest 0.1 cm and 0.1 kg, respectively.

### Fecal fat quantification

To assess the effect of the RODL formulation on fecal fat excretion, fecal samples were collected from participants at baseline (Day 2) and after the 2-day intervention period (Day 4). Participants were instructed to collect all feces excreted over a 24-h period at each time point using a standardized collection kit (including a fecal container, gloves, and cold packs), following detailed instructions provided by clinical staff. Samples were stored under refrigeration (2–8°C) immediately after collection and transported to the central laboratory within 24 h for analysis. Upon receipt, fecal samples were weighed and homogenized. For each sample, approximately 5 g of homogenized feces was subjected to lipid extraction using a modified Soxhlet extraction method with petroleum ether as the organic solvent. The dried fecal matter was placed into pre-weighed cellulose thimbles and extracted continuously with 150 mL of petroleum ether (boiling point: 40–60°C) for 4 h. Following extraction, the ether fraction was evaporated under reduced pressure, and the residual fat was dried and weighed gravimetrically. The amount of fecal fat was expressed in grams per day (g/day) based on the total wet weight of feces collected over 24 h.

### Biochemical assessments

To evaluate the metabolic safety and systemic effects of the RODL formulation, blood samples were collected at baseline (Day 2) and at the end of the intervention (Day 4) following overnight fasting for at least 10 h. Venous blood (approximately 10 mL) was drawn from the antecubital vein using standard aseptic technique and collected into serum-separating tubes (SST). Blood samples were drawn at baseline and endpoint for analysis of total cholesterol (TC), TG, high-density lipoprotein cholesterol (HDL-C), low-density lipoprotein cholesterol (LDL-C), aspartate aminotransferase (AST), alanine aminotransferase (ALT), gamma-glutamyl transferase (γ-GTP), and high-sensitivity C-reactive protein (hs-CRP). AEs and gastrointestinal symptoms were recorded daily.

### Safety assessments

Safety assessments were conducted to evaluate the tolerability of the RODL during the intervention period. AEs, clinical laboratory parameters, and vital signs were monitored throughout the study according to GCP guidelines. All participants were instructed to report any symptoms or adverse health events spontaneously or in response to open-ended questioning by clinical staff at each visit (Day 2: baseline, Day 4: post-intervention). Reported AEs were documented and classified according to the Medical Dictionary for Regulatory Activities (MedDRA) terminology. Each event was assessed for severity (mild, moderate, severe), duration, outcome, and its potential causal relationship to the investigational product (unrelated, possibly related, probably related, definitely related) by the study physician. Vital signs, including systolic and diastolic BP, heart rate, and body temperature, were measured in a seated position after 5 min of rest using a validated automated sphygmomanometer. Physical examinations were performed by qualified medical personnel at both visits to detect any clinically significant findings.

### Blinding procedures

To maintain double-blind conditions, the assignment codes linking participants to their respective groups (test or placebo) were securely sealed and managed exclusively by the principal investigator. Apart from information related to manufacturing, packaging, and labeling, the allocation details remained blinded throughout the study. Unblinding was permitted only in the event of a serious adverse drug reaction or other medically significant circumstances that necessitated identification of the intervention group for clinical decision-making. During the entire study period, no cases of unblinding occurred. Investigators supplied the test products to participants according to the randomized allocation numbers assigned at enrollment. In instances of product damage or loss, backup supplies with corresponding randomization codes were available and dispensed while strictly preserving blinding integrity. All study procedures, including emergency replacement of investigational products, were conducted following the double-blind protocol.

### Statistical analysis

All statistical analyses were performed using SAS^®^ software (version 9.4; SAS Institute Inc., Cary, NC, USA). The analysis was based on three predefined data sets: the Safety Set, FAS, and PPS. The Safety Set included all participants who received at least one dose of the investigational product and was used for safety evaluation. The FAS, defined according to the intention-to-treat (ITT) principle, included all randomized subjects who consumed at least one dose and had at least one post-baseline efficacy assessment, excluding those with major violations of inclusion or exclusion criteria. The PPS consisted of FAS participants who completed the study without major protocol deviations that could impact efficacy outcomes. The FAS was designated as the primary analysis set for efficacy, while the PPS was analyzed for supportive confirmation. Safety variables were analyzed in the Safety Set, and demographic and nutritional characteristics were assessed in the PPS. Missing values for efficacy endpoints were handled using the last observation carried forward (LOCF) method, except for pre-dose baseline values, which were not used for imputation. No imputation was performed for safety variables or other non-efficacy outcomes. Descriptive statistics were reported as mean ± SD. Between-group comparisons were conducted using two-sided tests with a significance level of α = 0.05. Normality of data distribution was assessed using the Shapiro–Wilk test. If both groups satisfied the normality assumption (*P* > 0.05), between-group comparisons were performed using an independent two-sample *t*-test. If normality was violated in either group, the Wilcoxon rank sum test was employed. In addition, for the analysis of change-from-baseline values, an analysis of covariance (ANCOVA) model was employed with baseline values as covariates to adjust for potential baseline differences. Least-squares means and 95% confidence intervals (CIs) were estimated, and *P*-values for between-group comparisons were reported from the ANCOVA model.

## Results

### Participant flow and baseline characteristics

**Fig. 2:**
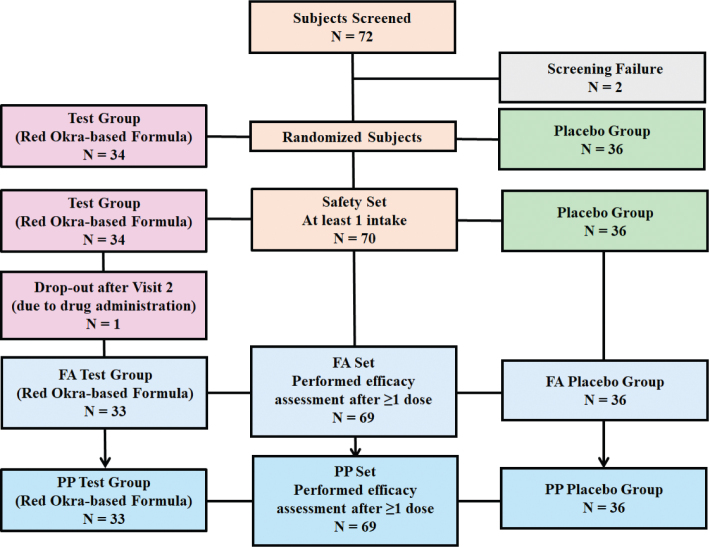
Flow diagram of subject enrollment, randomization, and analysis sets (FAS, PP) for the evaluation of the Red Okra-Diospyros lotus (RODL) formulation in healthy adults. A total of 72 subjects were screened, of whom 70 were randomized to receive either the RODL formulation (*N* = 34) or placebo (*N* = 36). All 70 participants were included in the Safety Set and Full Analysis Set (FAS) based on at least one dose administration and efficacy assessment. One participant in the RODL group discontinued due to non-compliance with the investigational product intake after Visit 2 and was excluded from the Per Protocol (PP) set. Thus, 69 participants completed the study per protocol and were included in the PP analysis (RODL group, *N* = 33; placebo group, *N* = 36).

A total of 72 individuals were screened for eligibility, and 70 participants who met the inclusion and exclusion criteria were randomized into either the RODL group (*n* = 34) or placebo group (*n* = 36). Among these, one participant in the RODL group was excluded from the FAS and PP analysis due to non-compliance with the investigational product intake on Day 2. Therefore, 69 subjects completed the study according to the protocol and were included in the FAS and PPS, while all 70 were included in the safety set (Fig. 2). Baseline demographic and clinical characteristics were comparable between the two groups (Table 2). The mean age was 30.33 ± 7.75 years in the RODL group and 27.75 ± 7.04 years in the placebo group (*P* = 0.2666). BMI at baseline was 25.26 ± 2.69 kg/m^2^ and 25.18 ± 2.68 kg/m^2^ in the RODL and placebo groups, respectively (*P* = 0.79). No statistically significant differences were observed between groups in sex distribution, bowel movement frequency, or other baseline parameters, confirming appropriate randomization.

**Table 2:** Baseline demographic and clinical characteristics of participants in the RODL and placebo groups

Variable		Placebo group	RODL group	*P*
Sex *N* (%)	*N*	36	33	1.000 (Fisher’s exact test)
	Male	32 (88.89%)	30 (90.91%)	
	Female	4 (11.11%)	3 (9.09%)	
Informed consent for fecal analysis *N* (%)		4 (100%)	3 (100%)	–
Age (years)		27.75 ± 7.04	30.33 ± 7.75	0.2666 (Wilcoxon)
Regular bowel habits (1–2 times/day), *N* (%)		36 (100%)	33 (100%)	–
Body weight (kg)		75.68 ± 11.17	75.42 ± 10.25	0.9213 (*t*-test)
Height (cm)		173.59 ± 5.86	173.35 ± 6.16	0.8704 (*t*-test)
BMI (kg/m^2^)		25.18 ± 2.68	25.26 ± 2.69	0.7915 (Wilcoxon)

Data are presented as mean ± SD or *n* (%). Statistical comparisons were conducted using Fisher’s exact test for categorical variables and Student’s *t*-test or Wilcoxon rank sum test for continuous variables, depending on normality. BMI: body mass index; RODL: red okra (*Abelmoschus esculentus*) and *Diospyros lotus*; SD: standard deviation.

### Validation of fecal matrix consistency for fecal fat analysis

**Fig. 3:**
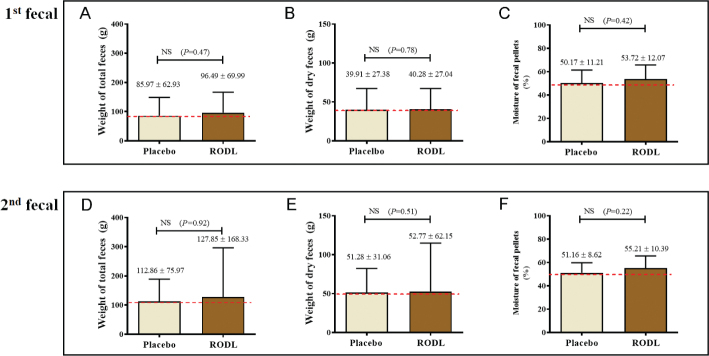
Assessment of fecal matrix consistency before and after RODL or placebo administration. (A–C) Baseline fecal characteristics measured on Day 2, including (A) wetfecal weight, (B) dry fecal weight, and (C) fecal moisture content, expressed as the percentage difference between wet and dry weights. (D–F) Fecal parameters re-assessed after the 2-day intervention on Day 4, including (D) wet fecal weight, (E) dry fecal weight, and (F) fecal moisture content. No statistically significant differences were observed between the placebo and RODL groups for any parameter at baseline or post-intervention (all *P* > 0.05). Intra-group comparisons from baseline to Day 4 also revealed no significant changes, confirming matrix stability. All data are presented as mean ± SD. Statistical significance was evaluated using independent two-sample *t*-tests or Wilcoxon rank-sum tests, depending on normality assumptions. *N* = 33 for RODL; *N* = 36 for placebo. NS: not significant; RODL: Red Okra-Diospyros lotus; SD: standard deviation.

To validate the consistency of the fecal matrix and ensure the reliability of fecal fat quantification, we examined whether the prescribed restricted diet and RODL administration influenced fecal parameters, including wet fecal weight, dry fecal weight, and fecal moisture content (Fig. 3). These parameters are known to affect gravimetric lipid quantification by altering sample dilution or introducing mass-based variability. At baseline (Day 2), the mean wet fecal weight was 85.97 ± 62.93 g in the placebo group and 96.49 ± 69.99 g in the RODL group, showing no statistically significant difference between groups (*P* > 0.05). After the 2-day intervention, fecal weights slightly increased to 112.86 ± 75.95 g (placebo) and 127.85 ± 168.33 g (RODL), but within-group changes from baseline were not statistically significant (*P* > 0.05) (Fig. 3A and D). Baseline dry fecal weights were also comparable between groups (39.91 ± 27.38 g vs. 40.28 ± 27.04 g; *P* > 0.05). Post-intervention values slightly increased to 51.28 ± 31.06 g (placebo) and 52.77 ± 62.15 g (RODL), but again, these within-group differences were not significant (*P* > 0.05) (Fig. 3B and E). Fecal moisture content, calculated as the relative difference between wet and dry weights, was 50.17 ± 11.21% in the placebo group and 53.72 ± 12.07% in the RODL group at baseline, with no significant intergroup difference (*P* > 0.05). Post-intervention moisture levels remained stable (51.16 ± 8.62% vs. 55.21 ± 10.39%, respectively), with no significant changes within groups (*P* > 0.05) (Fig. 3C and F). These results confirm that the fecal matrix was comparable between groups, and that fecal fat concentrations could be interpreted without the confounding effects of differential fecal mass or water content. The consistency in matrix parameters supports the validity of the observed differences in fecal fat output as being intervention-specific rather than artifactual.

### Fecal fat excretion

**Fig. 4:**
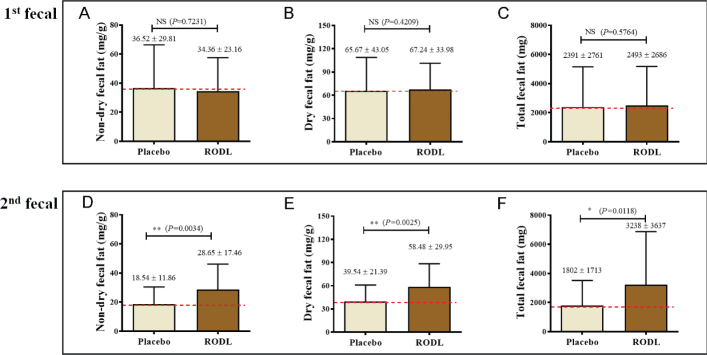
Effects of RODL on fecal fat excretion under a standardized restricted diet. Fecal fat content was analyzed in both placebo and RODL groups before (Day 2) and after (Day 4) a 2-day intervention using per-protocol (PP) analysis. Fecal samples were collected following a standardized restricted diet (130–150 g fat/day), and fecal lipids were quantified by gravimetric analysis after Soxhlet extraction. (A–C) Baseline fecal fat concentrations showed no significant difference between groups in wet fecal (A), dry fecal (B), and total fat mass (C). (D–F) Post-intervention analysis revealed a significant increase in fecal fat excretion in the RODL group compared to placebo, as measured by (D) fat content per gram of wet fecal, (E) per gram of dry fecal, and (F) total daily fat excreted in feces (mg/day). Red dotted lines represent the baseline mean values for each group. Data are presented as mean ± SD. Statistical comparisons were performed using the Wilcoxon rank-sum test and ANCOVA. *P < 0.05, **P < 0.01 vs. placebo. RODL: Red Okra-Diospyros lotus; SD: standard deviation; ANCOVA: analysis of covariance.

To evaluate the efficacy of RODL in promoting fecal fat excretion, fecal fat content was measured at baseline (Day 2) and after a 2-day intervention (Day 4) under a provided restricted diet (Fig. 4). Statistical analyses were performed using both the PP and FA sets. Comparisons included absolute values, changes from baseline (Δ), and between-group differences, with statistical significance determined using ANCOVA or Wilcoxon rank-sum tests, as appropriate. At baseline, wet fecal fat concentration did not significantly differ between the placebo group (36.52 ± 29.81 mg/g) and the RODL group (34.36 ± 23.16 mg/g) (*P* > 0.05; Fig. 4A). After intervention, the RODL group showed a significantly higher wet fecal fat concentration (28.65 ± 17.46 mg/g) than the placebo group (18.54 ± 11.86 mg/g; *P* = 0.0034, Wilcoxon; Fig. 4D). The change from baseline (Δ) was −17.98 ± 24.17 mg/g in the placebo group (*P* < 0.0001) and −5.71 ± 18.51 mg/g in the RODL group (*P* = 0.0858), and the between-group difference was statistically significant (*P* = 0.0417, Wilcoxon; *P* = 0.0004, ANCOVA). Baseline dry fecal fat concentrations were also comparable between groups (placebo: 65.67 ± 43.05 mg/g; RODL: 67.24 ± 33.98 mg/g; *P* > 0.05; Fig. 4B). Post-intervention, the RODL group exhibited a significantly higher dry fecal fat concentration (58.48 ± 29.95 mg/g) than the placebo group (39.54 ± 21.39 mg/g; *P* = 0.0025, Wilcoxon), although the within-group change in the RODL group was marginally non-significant (*P* = 0.0675; Fig. 4E). The Δ analysis showed −26.13 ± 33.76 mg/g in the placebo group (*P* < 0.0001) and −8.85 ± 26.86 mg/g in the RODL group (*P* = 0.0675), with a statistically significant between-group difference (*P* = 0.0417, Wilcoxon; *P* = 0.0004, ANCOVA). Similarly, baseline total fecal fat content (mg/day) was not significantly different between the placebo (2391.29 ± 2761.32 mg) and RODL (2493.18 ± 2685.62 mg) groups (*P* > 0.05; Fig. 4C). However, post-intervention measurements revealed a significantly greater total fecal fat output in the RODL group (3238.50 ± 3636.87 mg/day) compared to the placebo group (1801.67 ± 1712.77 mg/day; *P* = 0.0118, Wilcoxon; Fig. 4F). The Δ values were −589.62 ± 3057.46 mg/day in the placebo group (*P* = 0.2551) and +745.32 ± 3396.10 mg/day in the RODL group (*P* = 0.2165), and the between-group difference was statistically significant (*P* = 0.0331, ANCOVA).

### Fecal triglyceride excretion

**Fig. 5:**
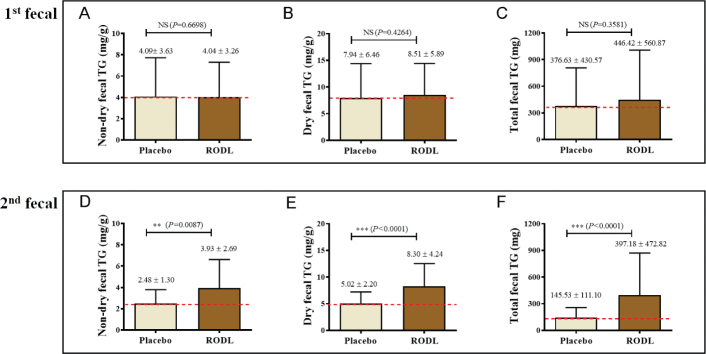
Effects of RODL on fecal triglyceride (TG) excretion under a standardized restricted diet. Fecal TG content was analyzed in both placebo and RODL groups before (Day 2) and after (Day 4) a 2-day intervention using per-protocol (PP) analysis. Fecal samples were collected following a standardized restricted diet (130–150 g fat/day), and fecal lipids were quantified by colorimetric enzymatic assay. (A–C) Baseline fecal TG concentrations showed no significant difference between groups in wet fecal (A), dry fecal (B), and total TG output (C). (D–F) Post-intervention analysis revealed a significant increase in fecal TG excretion in the RODL group compared to placebo, as measured by (D) TG concentration per gram of wet fecal, (E) per gram of dry fecal, and (F) total daily TG excreted in feces (mg/day). Red dotted lines represent the baseline mean values for each group. Data are presented as mean ± SD. Statistical comparisons were performed using the Wilcoxon rank-sum test and ANCOVA. *P < 0.05, **P < 0.01, ***P < 0.001 vs. placebo. RODL: Red Okra-Diospyros lotus; SD: standard deviation; ANCOVA: analysis of covariance.

To investigate the effect of RODL intake on fecal TG excretion, TG content in wet fecal, dry fecal, and total fecal output was assessed before (Day 2) and after (Day 4) a 2-day intervention under a provided restricted diet (Fig. 5). Statistical analyses were performed using both PP and FA sets. The comparisons included absolute values, changes from baseline (ΔTG), and between-group differences, with significance determined using ANCOVA and Wilcoxon rank-sum tests as appropriate. At baseline, no significant difference in wet fecal TG concentration was observed between the placebo group (4.09 ± 3.63 mg/g) and the RODL group (4.04 ± 3.26 mg/g, *P* > 0.05), indicating baseline equivalence (Fig. 5A). Following two administrations of RODL, the fecal TG concentration was significantly higher in the RODL group (3.93 ± 2.69 mg/g) than in the placebo group (2.48 ± 1.30 mg/g, *P* = 0.0087; Fig. 5D). The change from baseline (ΔTG) was –0.11 ± 3.08 mg/g in the RODL group and –1.61 ± 3.03 mg/g in the placebo group (*P* = 0.0015), indicating a statistically significant difference in treatment response. Baseline dry fecal TG concentrations were comparable between groups (placebo: 7.94 ± 6.46 mg/g; RODL: 8.51 ± 5.89 mg/g; *P* > 0.05; Fig. 5B). Post-intervention, the RODL group exhibited a significantly higher dry fecal TG concentration (8.30 ± 4.24 mg/g) than the placebo group (5.02 ± 2.20 mg/g, *P* < 0.0001; Fig. 5E). ΔTG was –0.21 ± 5.22 mg/g (RODL) vs. –2.92 ± 5.74 mg/g (placebo), showing a significant between-group difference (*P* < 0.0001). Total fecal TG excretion at baseline did not differ significantly between the placebo group (376.63 ± 430.57 mg) and the RODL group (446.42 ± 560.87 mg, *P* > 0.05; Fig. 5C). After the 2-day intervention, total TG output was significantly higher in the RODL group (397.18 ± 472.82 mg) than in the placebo group (145.53 ± 111.10 mg, *P* < 0.0001; Fig. 5F). The change in total TG excretion (ΔTG) also favored the RODL group (–49.23 ± 455.87 mg) compared to the placebo group (–231.11 ± 436.25 mg), resulting in a statistically significant between-group difference (*P* = 0.0022).

### Biochemical parameters

To investigate the systemic effects of RODL administration on lipid metabolism, serum concentrations of TC, HDL-C, LDL-C, TG, and FFA were measured at baseline and across three subsequent time points (Days 2–4) following daily intake under a standardized restricted diet (Table 3). At baseline, serum TC levels were comparable between the placebo (199.67 ± 27.87 mg/dL) and RODL (194.12 ± 33.95 mg/dL) groups (*P* > 0.05). Following the intervention, TC levels exhibited a gradual decline in both groups, reaching −11.21 ± 13.27 mg/dL in the RODL group by Day 4. However, no significant between-group differences were observed at any time point (all *P* > 0.05). Similarly, baseline HDL-C levels were not significantly different between the placebo and RODL groups (55.28 ± 14.73 vs. 50.33 ± 11.38 mg/dL, respectively). Both groups demonstrated a mild reduction in HDL-C during the intervention period, but the magnitude of change did not differ significantly between groups at any time point (Day 4: placebo: Δ = −1.39 ± 4.96 vs. RODL: Δ = −1.09 ± 5.40 mg/dL, *P* = 0.2548). Serum LDL-C levels were also comparable at baseline (placebo: 118.81 ± 27.93 mg/dL; RODL: 118.30 ± 32.22 mg/dL). A significant within-group decrease in LDL-C was observed in both groups by Day 4 (placebo: Δ = −3.50 ± 8.62 mg/dL, *P* = 0.0200; RODL: Δ = −5.52 ± 9.11 mg/dL, *P* = 0.0015), yet no statistically significant differences emerged between the groups (*P* > 0.05). Regarding serum TG levels, no differences were noted between groups at baseline. The placebo and RODL group showed a marked transient increase in TG following the Day 2 evening meal (placebo: 157.83 ± 172.40 mg/dL, *P* = 0.0021; RODL: 156.97 ± 95.12 mg/dL, *P* < 0.001) with no statistically significant differences between groups at any time point (all *P* > 0.05). By Day 4, TG levels in both groups returned to values close to baseline (placebo: 78.67 ± 54.87 mg/dL; RODL: 88.09 ± 48.30 mg/dL), indicating transient postprandial elevations with comparable recovery dynamics. Despite these fluctuations, TG levels remained statistically indistinguishable from those of the placebo group at all time points (*P* > 0.05). Baseline FFA levels were similar in both groups (placebo: 646.28 ± 311.18 μEq/L; RODL: 618.58 ± 221.28 μEq/L). A significant postprandial reduction in FFA was observed in both RODL and placebo group on Day 2 evening meal (placebo: Δ = -263.42 ± 461.62 μEq/L, *P* = 0.0016; RODL: Δ = -278.18 ± 209.05 μEq/L, *P* < 0.001), followed by a gradual return toward baseline levels on Days 2 and 3. Nonetheless, inter-group differences remained statistically non-significant throughout the study period.

**Table 3:** Effects of RODL administration on serum lipid profiles and free fatty acid levels over a 3-day intervention period

Parameter	Baseline		Day 2		Day 3		Day 4	
	Placebo group	RODL group	Placebo group	RODL group	Placebo group	RODL group	Placebo group	RODL group
Total cholesterol	199.67 ± 27.87	194.12 ± 33.95	185.08 ± 26.97	176.91 ± 30.58	186.69 ± 24.22	181.94 ± 33.80	189.06 ± 24.75	182.91 ± 31.07
Change from baseline			-14.58 ± 10.18	-17.21 ± 13.45	-12.97 ± 9.35	-12.18 ± 15.38	-10.61 ± 11.22	-11.21 ± 13.27
HDL-cholesterol	55.28 ± 14.73	50.33 ± 11.38	50.39 ± 13.60	44.73 ± 10.97	52.50 ± 12.71	48.48 ± 11.16	53.89 ± 11.87	49.24 ± 10.44
Change from baseline			-4.89 ± 3.28	-5.61 ± 4.64	-2.78 ± 4.59	-1.85 ± 5.75	-1.39 ± 4.96	-1.09 ± 5.40
LDL-cholesterol	118.81 ± 27.93	118.30 ± 32.22	109.11 ± 25.24	107.00 ± 26.80	111.72 ± 25.49	110.70 ± 30.45	115.31 ± 25.99	112.79 ± 30.33
Change from baseline			-9.69 ± 6.42	-11.30 ± 9.06	-7.08 ± 7.28	-7.61 ± 10.55	-3.50 ± 8.62	-5.52 ± 9.11
Triglyceride	96.78 ± 76.35	103.00 ± 51.09	157.83 ± 172.40	156.97 ± 95.12	125.69 ± 105.85	132.36 ± 81.34	78.67 ± 54.87	88.09 ± 48.30
Change from baseline			61.06 ± 110.25	53.97 ± 61.55	28.92 ± 71.26	29.36 ± 56.86	-18.11 ± 31.31	-14.91 ± 35.79
FFA	646.28 ± 311.18	618.58 ± 221.28	382.86 ± 390.37	340.39 ± 170.45	275.83 ± 126.23	285.12 ± 151.90	394.69 ± 116.91	413.21 ± 143.89
Change from baseline			-263.42 ± 461.62	-278.18 ± 209.05	-370.44 ± 274.35	-333.45 ± 224.92	-251.58 ± 307.67	-205.36 ± 241.21

Serum levels of total cholesterol (TC), HDL-cholesterol (HDL-C), LDL-cholesterol (LDL-C), triglycerides (TG), and free fatty acids (FFA) were measured at baseline and at Days 2, 3, and 4 following intervention under a standardized fat-restricted diet (130–150 g/day). Values are expressed as mean ± SD. ‘Change from baseline’ indicates the difference from Day 0 for each parameter. No significant between-group differences were observed at any time point (all *P* > 0.05). Total Cholesterol, HDL-Cholesterol, LDL-Cholesterol, and Triglyceride unit: mg/dL; FFA unit: μEq/L. Statistical analysis was conducted using ANCOVA and Wilcoxon rank-sum tests. RODL: red okra (*Abelmoschus esculentus*) and *Diospyros lotus*; SD: standard deviation; ANCOVA: analysis of covariance.

### Safety assessment

Safety analyses were conducted using the Safety Set population, which comprised all participants who consumed the investigational product at least once after randomization (*n* = 34 in the RODL group; *n* = 36 in the placebo group). Safety endpoints included the incidence, severity, and causality of AEs, as well as evaluations of blood chemistry, vital signs (blood pressure and heart rate), anthropometric measurements (body weight), and electrocardiography (ECG). A total of three AEs were reported in two participants (2.78%) in the placebo group, while one AE occurred in one participant (2.94%) in the RODL group. All AEs were mild in severity, transient, and assessed as unrelated to the investigational product. No serious AEs or study discontinuations due to AEs were observed during the study period. Laboratory analyses revealed no significant differences between groups in blood chemistry parameters following the 3-day intervention (Table 4). Heart rate measurements showed a statistically significant difference between groups on Day 2; the placebo group demonstrated a mean change of −1.25 ± 10.78 beats/min, and the RODL group showed a reduction of −5.97 ± 8.47 beats/min (*P* = 0.0485). However, no between-group difference was observed on Day 3, and all observed changes remained within the clinically acceptable normal range. No significant between-group differences were detected in systolic or diastolic blood pressure or body weight on Day 3 (Table 5). Additionally, no meaningful changes were observed in ECG parameters before and after the intervention in either group (data not shown). Serum biochemical analyses performed at baseline and post-intervention included AST, ALT, γ-GTP, creatinine, and BUN. No statistically significant between-group differences were observed, and all measured values remained within normal clinical ranges during the study period, indicating no evidence of hepatotoxicity or nephrotoxicity associated with short-term RODL administration. Collectively, these findings demonstrate that short-term administration of RODL was safe and well tolerated, with no clinically significant safety concerns arising during the study.

**Table 4:** Changes in serum biochemical parameters following 2-day intervention with RODL or placebo under a restricted diet

Parameter	Baseline		Day 2	
	Placebo group	RODL group	Placebo group	RODL group
AST(GOT)(U/L)	24.97 ± 10.45	21.85 ± 5.46	23.97 ± 28.13	19.15 ± 5.21
Change from baseline			-1.00 ± 22.19	-2.73 ± 3.62
ALT(GPT)(U/L)	25.53 ± 17.83	23.88 ± 16.52	23.56 ± 19.36	22.33 ± 17.85
Change from baseline			-1.97 ± 9.99	-1.61 ± 4.49
γ-GTP(U/L)	27.19 ± 25.64	27.82 ± 18.90	25.00 ± 22.29	25.27 ± 16.94
Change from baseline			-2.19 ± 4.48	-2.27 ± 3.04
Creatinine(mg/dL)	0.91 ± 0.12	0.89 ± 0.14	0.93 ± 0.13	0.92 ± 0.14
Change from baseline			0.02 ± 0.08	0.03 ± 0.10
BUN(mg/dL)	12.53 ± 2.56	13.56 ± 2.77	15.83 ± 2.48	16.88 ± 2.56
Change from baseline			3.31 ± 2.30	3.45 ± 2.67

Serum levels of aspartate aminotransferase (AST), alanine aminotransferase (ALT), γ-glutamyl transpeptidase (γ-GTP), creatinine, and blood urea nitrogen (BUN) were measured at baseline (Day 1) and on Day 2 after intervention. Data are presented as mean ± SD. No statistically significant differences were observed between groups at either time point. All measured parameters remained within normal clinical reference ranges, indicating no hepatotoxic or nephrotoxic effects associated with short-term RODL administration.

AST, ALT, and γ-GTP are expressed in U/L; creatinine and BUN are expressed in mg/dL. RODL: red okra (*Abelmoschus esculentus*) and *Diospyros lotus*; SD: standard deviation.

**Table 5:** Changes in vital signs and body weight following short-term intervention with RODL or placebo

Parameter	Baseline		Day 2		Day 3	
	Placebo group	RODL group	Placebo group	RODL group	Placebo group	RODL group
SBP (mmHg)	123.78 ± 9.82	121.82 ± 11.48	125.33 ± 6.32	121.88 ± 10.18	125.44 ± 7.77	121.45 ± 9.04
Change from baseline			1.56 ± 8.31	-0.27 ± 8.06	1.67 ± 9.48	-0.70 ± 10.52
DBP (mmHg)	72.81 ± 11.52	75.29 ± 11.79	73.83 ± 10.00	73.45 ± 13.30	76.17 ± 9.69	76.73 ± 10.19
Change from baseline			1.03 ± 6.95	-1.79 ± 9.16	3.36 ± 8.48	1.48 ± 9.84
Heart rate (beats/min)	77.28 ± 13.24	80.26 ± 10.92	76.03 ± 10.55	73.79 ± 9.88	76.19 ± 12.26	76.06 ± 10.64
Change from baseline			-1.25 ± 10.78	-5.97 ± 8.47*	-1.08 ± 11.33	-3.70 ± 8.42
Body Weight (kg)	75.68 ± 11.17	75.29 ± 10.12	75.81 ± 11.05	75.56 ± 10.35	75.19 ± 11.07	75.25 ± 10.31
Change from baseline			0.14 ± 0.56	0.14 ± 0.63	-0.48 ± 0.71	-0.18 ± 0.89

Systolic blood pressure (SBP), diastolic blood pressure (DBP), heart rate, and body weight were assessed at baseline, Day 2, and Day 3 after intervention under a restricted dietary protocol. Data are presented as mean ± SD. A statistically significant reduction in heart rate was observed in the RODL group on Day 2 compared to the placebo group (*P = 0.0485). However, these changes remained within the normal clinical range and were not considered clinically significant. No significant between-group differences were observed in SBP, DBP, or body weight at any time point. SBP and DBP are expressed in mmHg, heart rate in beats/min, and body weight in kg. RODL: red okra (*Abelmoschus esculentus*) and *Diospyros lotus*; SD: standard deviation.

## Discussion

In light of the global obesity epidemic, which is largely driven by excessive dietary fat intake and sedentary lifestyles, the development of safe, food-derived strategies to modulate lipid metabolism has gained increasing clinical relevance (15). Fecal fat excretion is one of the most direct indicators of reduced intestinal lipid absorption and has emerged as a promising target for nutritional interventions aimed at managing body fat accumulation (16). However, despite the well-documented fat-binding properties of certain dietary fibers and phytochemicals, few botanical formulations have demonstrated statistically significant fat-excreting efficacy in controlled human clinical trials. Furthermore, in light of the global obesity epidemic, which is largely driven by excessive dietary fat intake and sedentary lifestyles, the development of safe, food-derived strategies to modulate lipid metabolism has gained increasing clinical relevance (15). Fecal fat excretion is one of the most direct indicators of reduced intestinal lipid absorption and has emerged as a promising target for nutritional interventions aimed at managing body fat accumulation (16). However, despite the well-documented fat-binding properties of certain dietary fibers and phytochemicals, few botanical formulations have demonstrated statistically significant fat-excreting efficacy in controlled human clinical trials.

To address this gap, the present randomized, double-blind, placebo-controlled human study was conducted to evaluate the fat-excreting efficacy and clinical safety of a botanical formulation composed of red okra fruit and *D. lotus* leaf, herein referred to as RODL. The clinical trial was conducted under a strictly standardized dietary protocol (130–150 g/day of fat) to control for confounding variation in baseline fat intake, a major methodological advancement compared to previous studies, which lacked diet standardization (17).

Previous *in vitro* and animal studies demonstrated that this formulation inhibits adipocyte differentiation and lipid accumulation, and promotes gastrointestinal lipid excretion by interfering with lipid absorption mechanisms (13). Consistent with these preclinical findings, our human intervention trial confirmed a statistically significant increase in total fecal fat excretion (dry weight basis) in the RODL group compared to the placebo group after two consecutive days of administration under a provided restricted diet. The enhanced fecal fat output occurred without significant alterations in fecal weight or moisture content, indicating that the fat-binding effect was not confounded by changes in fecal matrix properties. The intergroup difference in fecal fat mass (Δg) was most pronounced on Day 4, indicating that the RODL formulation enhances gastrointestinal lipid elimination. These findings are consistent with preclinical studies showing that RODL physically binds dietary fat and impairs lipid micelle formation via its mucilage and polyphenol constituents. This fat-excreting action is presumed to result from physical fat binding by soluble fiber components and the emulsification-inhibitory action of polyphenols and flavonoids present in both red okra and *D. lotus* leaves (6, 9).

While serum lipid profiles, including TC, LDL-C, and TG, did not show statistically significant between-group differences, a consistent trend toward lipid reduction was observed in the RODL group across multiple time points. Notably, the reduction in LDL-C within the RODL group reached statistical significance by Day 4 (*P* = 0.0015), despite the short intervention duration. This trend suggests a potential systemic lipid-lowering effect of RODL, which may be mediated through the modulation of intestinal lipid absorption and hepatic lipid metabolism, as shown in previous animal models (13). Furthermore, these findings may suggest a dual mechanism wherein RODL not only binds lipids in the gastrointestinal tract but also modestly modulates systemic lipid metabolism, possibly via hepatic or intestinal farnesoid X receptor (FXR)-related pathways, as previously reported in polyphenol-rich interventions (18, 19).

Safety outcomes were favorable. Only one mild AE was reported in the intervention group, and no SAEs or discontinuations due to AEs were observed. Vital signs, ECGs, and blood chemistry values remained within normal limits. The only statistically significant difference was a modest reduction in heart rate in the RODL group on Day 2 (*P* = 0.0485), which was not considered clinically significant and resolved by Day 3. These data collectively support the safety and tolerability of RODL for short-term administration. Prior to initiation of the clinical study, the RODL formulation underwent comprehensive GLP-compliant safety evaluation and quality-control assessment as part of a separate project on formulation safety and stability. The final clinical sample was also assessed for safety-related quality parameters, including heavy metals and other potentially concerning residues. Because these data are being reported separately in an independent safety-focused study, the present manuscript focuses on clinical efficacy and tolerability outcomes.

Taken together, the findings of this study suggest that short-term supplementation with RODL under controlled dietary conditions can enhance fecal fat excretion without compromising systemic safety. While the lipid-lowering effects observed in the bloodstream were not statistically conclusive, the consistent trends observed across multiple lipid parameters provide a rationale for further investigation in longer-term or hyperlipidemic cohorts. Future studies should explore dose optimization, mechanistic validation through metabolomic profiling, and long-term safety evaluations in populations with metabolic disorders. The findings of this study hold particular value given the limitations of existing pharmacologic fat blockers such as orlistat, which are often associated with adverse gastrointestinal effects and poor long-term compliance (11, 20). RODL offers a food-grade alternative that combines efficacy with excellent tolerability, making it a promising candidate for preventive strategies targeting postprandial lipid absorption.

This study provides clinical validation for the fat-binding effect of a botanical RODL combination, as evidenced by enhanced fecal lipid excretion in healthy adults. The increase in fecal fat output suggests that the extract may reduce intestinal lipid absorption, thus reducing dietary fat availability for systemic storage.

The findings are consistent with previous animal studies demonstrating fecal TG elevation and weight gain suppression following RODL administration. Mechanistically, RO is likely responsible for mucilage-mediated fat sequestration, while DL contributes polyphenol-driven metabolic modulation. The clinical safety and efficacy data presented here underscore the potential of RODL as a functional ingredient for managing dietary fat overload and supporting weight control. Nevertheless, the current study is subject to several limitations. First, the short intervention period (2 days of dosing and 3-day follow-up) may have limited the observable effects on systemic lipid markers. Second, while dietary control was implemented, gut microbiota composition, which may influence lipid metabolism and fecal excretion, was not assessed. Third, the molecular mechanisms underlying fat-binding and metabolic modulation were not investigated in this trial and should be further explored using multi-omics approaches. To establish the long-term clinical utility of RODL, future trials should evaluate chronic administration over multiple weeks, assess changes in body composition, and explore mechanistic biomarkers such as bile acid profiles, gut microbial shifts, and hepatic gene expression. Moreover, dose-response studies and pharmacokinetic evaluations of the active constituents, such as quercetin-3-O-gentiobioside and gallic acid, may provide critical insights into optimal formulation design.

## Conclusion

This randomized, double-blind, placebo-controlled clinical trial provides the first human evidence that a botanical formulation composed of RODL™ significantly enhances fecal fat and TG excretion under a standardized restricted fat diet. By implementing rigorous dietary control and validated gravimetric fat quantification protocols, the study isolated the fat-sequestering effects of RODL from confounding dietary or fecal matrix variables. The intervention produced significant increases in fecal fat output without affecting fecal hydration, systemic lipid homeostasis, or safety parameters. Importantly, the observed effects were achieved without adverse gastrointestinal symptoms, distinguishing RODL from conventional pharmacologic fat blockers. The botanical formulation was well tolerated, with no SAEs or clinically meaningful changes in vital signs, ECG, or blood chemistry. These findings highlight RODL as a safe and effective dietary intervention capable of physically reducing intestinal fat absorption, thus offering a promising adjunctive strategy for individuals seeking dietary fat regulation or obesity prevention through food-based approaches. Future long-term studies are warranted to evaluate sustained effects on body weight, metabolic health, and adiposity-related endpoints, as well as to explore the underlying molecular mechanisms.

## Data Availability

All data generated or analyzed during this study are included in this published article.
